# Accurate Wide Angle SAR Imaging Based on LS-CS-Residual

**DOI:** 10.3390/s19030490

**Published:** 2019-01-25

**Authors:** Zhonghao Wei, Bingchen Zhang, Yirong Wu

**Affiliations:** 1 Key Laboratory of Technology in Geo-Spatial Information Processing and Application System, Institute of Electronics, Chinese Academy of Sciences, Beijing 100190, China; bczhang@mail.ie.ac.cn (B.Z.); wyr@mail.ie.ac.cn (Y.W.); 2 School of Electronic, Electrical and Communication Engineering, University of Chinese Academy of Sciences, Beijing 101408, China; 3 Institute of Electronics, Chinese Academy of Sciences, Beijing 100190, China

**Keywords:** wide angle SAR, compressed sensing, LS-CS-Residual, aspect dependent

## Abstract

Wide angle synthetic aperture radar (WASAR) receives data from a large angle, which causes the problem of aspect dependent scattering. $L_{1}$ regularization is a common compressed sensing (CS) model. The $L_{1}$ regularization based WASAR imaging method divides the whole aperture into subapertures and reconstructs the subaperture images individually. However, the aspect dependent scattering recovery of it is not accurate. The subaperture images of WASAR can be regarded as the SAR video. The support set among the different frames of SAR video are highly overlapped. Least squares on compressed sensing residuals (LS-CS-Residuals) can reconstruct the time sequences of sparse signals which change slowly with time. This is to replace CS on the observation by CS on the least squares (LS) residual computed using the prior estimate of the support. In this paper, we introduce LS-CS-Residual into WASAR imaging. In the iteration of LS-CS-Residual, the azimuth-range decoupled operators are used to avoid the huge memory cost. Real data processing results show that LS-CS-Residual can estimate the aspect dependent scatterings of the targets more accurately than CS based methods.

## 1. Introduction

Wide angle synthetic aperture radar (WASAR) receives echo data from a large angle. Advances in synthetic aperture radar (SAR) technology have enabled coherent sensing of WASAR. Circular SAR (CSAR) is a specific case of WASAR whose track is circular. With the increase of the synthetic angle, because of the reflector geometry, shadowing, and coherent scintillation, the problem of aspect dependent scattering [1,2] arises. Traditional imaging methods are based on the isotropic assumption. It means that the scattering is constant in the synthetic aperture angle, which is not valid in WASAR.

To accommodate the aspect dependent scattering, there are mainly two approaches, the subaperture approach and full aperture approach [2]. The subaperture approach [1] divides the whole aperture into the subapertures and assumes that the scatterings are constant in the subaperture. Then, the narrow angle imaging methods such as matched filtering [3] and an $L_{1}$ regularization based SAR imaging method [4,5] can be adopted for the subaperture imaging. For the full aperture approach, they can be divided into two kinds. The first one assumes that the scattering during one subaperture is isotropic and reconstructs an imaging model with all subapertures included [6,7,8]. The subaperture images are recovered jointly. The other one is the parametric method [9,10]. It assumes that the scene includes some scattering targets and that their scatterings follow some functions. The scattering functions of the targets are fitting with the whole aperture data included.

In the past decade, compressed sensing (CS) [11,12] has drawn much attention in sparse signal processing, which provides reconstruction guarantees for sparse solutions to linear inverse problems. It is shown that, when the scene is sparse and the measurement matrix satisfies the Restricted Isometry Property (RIP), the signal can be recovered with down-sampled data by solving an $L_{1}$ minimization problem [12]. An $L_{1}$ minimization problem is also known as Basis Pursuit. The theory of Lagrange multipliers indicates that we can solve an unconstrained problem that will yield the same solution, provided that the Lagrange multipler is selected correctly. The unconstrained problem is known as LASSO or $L_{1}$ regularization [13]. For the subaperture reconstruction based on $L_{1}$ regularization, it mainly has two drawbacks. Firstly, as a common reconstructed model in compressed sensing (CS), $L_{1}$ regularization is a biased estimator [14], which means that the amplitude of the targets would be underestimated. Secondly, the support set of the targets is not accurately estimated with the data of one subaperture and there are some missed detections. For the first drawback, it can be solved via debiased-CS proposed in [15,16]. Debiased-CS is a two-step method, which firstly reconstructs the signal with CS and calculates the least squares (LS) estimates on the support set of the signal. For the second drawback, since the support sets of different aspect subaperture images are highly overlapped across the whole aperture [9], this information can be adopted in the subaperture image reconstruction to avoid it.

The idea of CS is to compressively sense signals that are sparse in some known domains and then use sparse recovery techniques to recover them. Considering the dynamic CS problem, i.e., the problem of recovering a time sequence of sparse signals, CS recovers each sparse signal in the sequence independently without using any information from other frames. Least squares on compressed sensing residual (LS-CS-Residual) [17] is to replace CS on the observation by CS on the least squares (LS) residual computed using the prior estimate of the support. It is suitable for dynamic CS problem. It is proved that LS-CS-Residual can recover the signal better than CS [17].

The subaperture images of WASAR can be regarded as the SAR video. Every frame is the subaperture image indexed by the aspect angle. In WASAR, the backscattering from a complex target at high frequencies can be approximately modeled as a discrete set of the scattering centers [9].The scattering center can be described by the aspect-dependent amplitude and position [9]. The supports of the scattering centers overlap across the whole aperture. This information can be adopted in WASAR imaging.

In this paper, we propose a novel subaperture imaging method based on LS-CS-Residual. The proposed method firstly implements Backprojection (BP) on all of the aperture data. Then, the coarse support set is estimated from the BP image. For every subaperture of WASAR, the least squares estimate on the support set is calculated. Then, the observation residual is calculated. With the residual data, we can solve the residual observation model with $L_{1}$ regularization. The accurate supports of subaperture images are estimated from the $L_{1}$ regularization image. Finally, the LS estimate on the accurate supports is calculated. Since the structure information and LS estimate on the support set are adopted in the proposed method, it can recover the aspect dependent scattering more accurately than CS and debiased-CS.

In the iteration of LS-CS-Residual, there are matrix-vector products. For large scale scenes, the storage of measurement matrix can cost a huge amount of memory. A common strategy is to adopt the azimuth-range decouple operators in the algorithm. In this paper, the BP based azimuth-range decouple operators are adopted. The memory cost is reduced from $O(n^{2})$ to O(n).

This paper is organized as follows. In Section 2, we describe the WASAR subaperture imaging model based on CS. Section 3 introduces the WASAR imaging method based on LS-CS-Residual. Section 4 presents the experimental results. The conclusions are presented in Section 5.

## 2. WASAR Subaperture Imaging Based on Compressed Sensing

**Figure 1:**
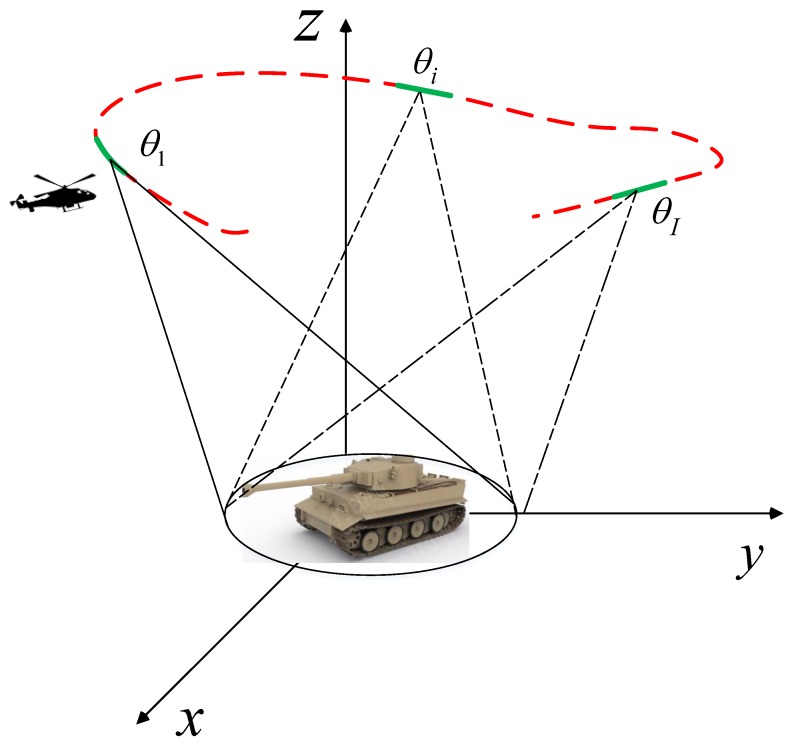
The configuration of WASAR.

WASAR receives data from a large angle. The configuration of WASAR is depicted in Figure 1. The whole aperture can be divided into subapertures. For the data collected from a little subaperture, its scattering can be regarded as constant. Then, the phase history of the *i*-th (i=1,2⋯I) subaperture is formulated as

(1) $$\begin{array}{c} r_{i}\left(f_{p},\theta_{q}\right)=\sum_{m=1}^{M}\sum_{n=1}^{N}s_{i}\left(x_{m},y_{n}\right)\cdot \exp\left\{-j\frac{4\pi f_{p}}{c}\cdot \left(x_{m}\cos\left(\theta_{q}\right)+y_{n}\sin\left(\theta_{q}\right)\right)\right\}+z_{i}, \end{array}$$

where $r_{i}$ is the phase history data of the *i*-th subaperture, and *m* and *n* are the pixel indexes along *x* and *y*. *M* and *N* are the pixel numbers along *x* and *y*, *s* is the scattering reflectivity of the *i*-th subaperture which is located at $\left(x_{m},y_{n}\right)$, $f_{p}\;\left(p=1,2\cdots P\right)$ is the frequency, *c* is the light velocity, $\theta_{q}\;\left(q=1,2\cdots Q\right)$ is the aspect angle, and $z_{i}$ is additive noise.

We vectorize Equation (1) and express it in a compact form

(2) $$r_{i}=\Phi_{i}\cdot s_{i}+z_{i},$$

where $r_{i}$ is the history data of *i*-th subaperture, $s_{i}$ is the backscattering of *i*-th subaperture, and $z_{i}$ is the noise, the measurement matrix $\Phi_{i}$ is shown as

(3) $$\Phi_{i}=\left[\begin{array}{cccc} \phi_{i}\left(1,1\right) & \phi_{i}\left(1,2\right) & \ldots & \phi_{i}\left(1,MN\right)\\ \vdots & \vdots & \ddots & \vdots \\ \phi_{i}\left(pq,1\right) & \phi_{i}\left(pq,2\right) & \ldots & \phi_{i}\left(pq,MN\right)\\ \vdots & \vdots & \ddots & \vdots \\ \phi_{i}\left(PQ,1\right) & \phi_{i}\left(PQ,2\right) & \ldots & \phi_{i}\left(PQ,MN\right) \end{array}\right],$$

where $\phi_{i}\left(pq,mn\right)=\exp\left\{-j\frac{4\pi f_{p}}{c}\left(x_{m}\cos\left(\theta_{q}\right)+y_{n}\sin\left(\theta_{q}\right)\right)\right\}$.

The subaperture imaging methods for WASAR imaging assume that the scattering of the targets are not relevant to the aspect angle in a narrow angle. Then, a traditional imaging method can be implemented in subaperture image focusing.

CS has been introduced into SAR imaging [4]. When the scene is sparse and the measurement matrix satisfies the restricted isometry property (RIP) condition, Equation (2) can be solved via $L_{1}$ regularization [18]

(4) $$\min_{s_{i}}∥r_{i}-\Phi_{i}\cdot s_{i}{∥}_{2}^{2}+\lambda {∥s_{i}∥}_{1}.$$

where λ is the regularization parameter.

For Equation (4), the optimality condition is

(5) $$2\Phi_{i}^{H}\left(\Phi_{i}s_{i}-r_{i}\right)+\lambda p=0,$$

where ${\left(\cdot \right)}^{H}$ is the conjugate transpose and

(6) $$p=\partial ∥s_{i}{∥}_{1}.$$

Suppose the oracle support of the $s_{i}$ is *T*, and then the solution of (4) is

(7) $${\left(s_{i}\right)}_{T}=\Phi_{i}{}_{T}^{†}r_{i}-\lambda {\left(\Phi_{i}{}_{T}^{H}\Phi_{iT}\right)}^{-1}\mathrm{sign}\left(s_{iT}\right),{\left(s_{i}\right)}_{{}_{T^{C}}}=0,$$

where $\Phi_{i}{}_{T}^{†}={\left(\Phi_{i}{}_{T}^{H}\Phi_{i}{}_{T}\right)}^{-1}\Phi_{i}{}_{T}^{H}$, $T^{C}$ denotes the complement of *T*. sign(·) is the signal function formulated as

(8) $$\mathrm{sign}\left(s_{i}\right)=\frac{s_{i}}{|s_{i}|}.$$

If the oracle support is accurate, then the first term of (7) is the exact estimate of the signal. The second term of (7) is the bias that is brought by the regularized term of (4).

In [14], it is shown that $L_{1}$ can reconstruct the targets with the underestimated amplitudes. Some missed detections are also introduced in the results of $L_{1}$ regularization. In addition, with less azimuth measurements, the resolution of the subaperture is reduced. The underestimation can be reduced via LS on the support. The missed detections can be reduced when more information is adopted. In the next section, we will propose a novel method for WASAR subaperture imaging.

## 3. WASAR Imaging Based on LS-CS-Residual

$L_{1}$ regularization would cause the errors of the amplitude and support set estimation in WASAR imaging. In this section, we propose a novel WASAR imaging based on LS-CS-Residual.

In WASAR, the subaperture images can be regarded as the video indexed on subaperture [2], which is a map of reflectivity as a function of viewing angle. The reflectivities of the targets can be described via their amplitudes and positions. Although they vary with aspect angle, the positions are highly overlapped. Some methods for dynamic scene such as video signal processing and dynamic MRI imaging can be introduced to WASAR imaging.

LS-CS-Residual [17] has been proposed for dynamic CS problems, such as dynamic magnetic resonance imaging (MRI). The idea of LS-CS-Residual is to perform CS not from the observations, but from the least squares residual computed using the previous support estimation. It is shown that it needs fewer samples and the bounded reconstruction error is smaller than the traditional CS. In the model of LS-CS-Residual, the information between different frames are used and there is also a debiasing step in the final to reduce the bias caused by $L_{1}$ regularization. It can reconstruct the results much more accurately than CS.

Since the support sets of between different WASAR subaperture images are highly overlapped, which means that WASAR imaging can be regarded as a dynamic problem. Thus, LS-CS-Residual is suitable for WASAR subaperture imaging. In the frame of LS-CS-Residual, the LS estimate is included, which means that the underestimation of $L_{1}$ regularization is avoided. In addition, the support information of different subapertures will be used, which will make the results more accurate.

LS-CS-Residual mainly has three steps: initial LS estimation, implementing CS on the residual (CS-Residual) and final LS estimate.   

**Initial LS Estimate**   

For Equation (2), if the support set of $s_{\theta_{i}}$ is known, we could simply compute the LS estimate on the support while setting all other values to zeros. The previous support can be estimated from the prior information. Suppose the estimated support is *T*, to compute and initial LS estimate

(9) $${\left(s_{i,init}\right)}_{T}={\left(\Phi_{i}T\right)}^{†}r_{i},{\left(s_{i,init}\right)}_{T^{c}}=0.$$

Then, the LS residual is calculated as

(10) $$r_{i,res}=r_{i}-\Phi_{i}s_{i,init}.$$

In WASAR, the scattering of the targets is aspect dependent. However, the support sets of the subaperture images are highly overlapped, which means that a fairly accurate support *T* can be estimated from the data. *T* is estimated via

(11) $$T=supp(s_{0}:|s_{0}|>\alpha ),$$

which is the support of the elements whose amplitudes are larger than α.

In [17], the threshold α is determined by the *b*%-Energy support, which means that *T* contains at least b% of the signal energy. In WASAR imaging, we set b%=90%.

Notice that the LS residual, $r_{i,res}$, can be rewritten as

(12) $$r_{i,res}=\Phi_{i}\beta_{i},\beta_{i}=s_{i}-s_{i,init}.$$

**CS-Residual**  

In this step, CS is implemented on the LS residual, i.e., solve (12) with CS in the following model

(13) $$\min_{\beta_{i}}∥r_{i,res}-\Phi_{i}\beta_{i}{∥}_{2}^{2}+\lambda {∥\beta_{i}∥}_{1}.$$

Iterative shrinkage thresholding algorithm (ISTA) [19] can be used to solve (13). In the iteration of ISTA, there are no matrix inversions involved. ISTA is preferred for its simplicity in implementation for distributed or parallel recovery due to nature of the involved matrix-vector multiplications [20,21]. The iteration is formulated as

(14) $$\hat{\beta_{i}^{t}}=\beta_{i}^{t}+\mu \left[\Phi_{i}^{H}\left(r_{i}-\Phi_{i}\beta_{i}^{t}\right)\right],$$

(15) $$\beta_{i}^{t+1}=f_{\lambda \mu }\left(\hat{\beta_{i}^{t}}\right)=\left\{\begin{array}{cc} \mathrm{sgn}\left(\hat{\beta_{i}^{t}}\right)\left(\right|\hat{\beta_{i}^{t}}\left|-\lambda \mu \right), & \mathrm{if}\;|\hat{\beta_{i}^{t}}|>\lambda \mu \\ 0, & \mathrm{otherwise}, \end{array}\right.$$

where $\mu \in (0,∥A{∥}_{2}^{-2})$ is the step size controlling the convergence, λ is the regularization parameter, and *f* is the iterative function of ISTA. In the iteration, the value of λ is

(16) $$\lambda =|\hat{\beta_{i}^{t}}{|}_{K+1}/\mu ,$$

where $|\hat{\beta_{i}^{t}}{|}_{K+1}$ is the (K+1)-th largest element of $\hat{\beta_{i}^{t}}$ and $K=∥\hat{\beta_{i}^{t}}{∥}_{0}$.

The final estimation is

(17) $$\hat{s_{i}}=\beta_{i}+s_{i,\mathrm{init}}.$$

**Final LS Estimation**   

It is shown that $\beta_{i}$ is obtained after $L_{1}$ regularization, and the estimation will be biased towards zeros. Thus, a debiasing step is needed

(18) $$T^{\prime }=\mathrm{supp}\left(\hat{s_{i}}\right),$$

(19) $$s_{i}{}_{T}={\left(\Phi_{i}{}_{T^{\prime }}\right)}^{†}r_{i},s_{i}{}_{{T^{\prime }}^{C}}=0.$$

After the construction of the subaperture images, the generalized likelihood ratio test (GLRT) [1] can be implemented for the final composite image. GLRT is defined as

(20) $$s\left(x,y\right)=\max_{i}\left|s^{i}\left(x,y\right)\right|,$$

where $s^{i}\left(x,y\right)$ is the scattering at pixel (x,y) of *i*-th subaperture.

The algorithm is summarized in Algorithm 1.

**Algorithm 1** LS-CS-Residual based WASAR imaging.
**Input:** Subaperture echo data $r_{i}\;\left(i=1:I\right)$ and measurement matrix $\Phi_{i}$, iterative parameter μ, maxmum iterative step $T_{\max}$. 1:Implement BP on the whole aperture data, estimate *T* from the BP image. $s_{i}=0\;\left(i=1:I\right)$, t=0. 2:
**for** i=1:I **do**
 3: ${\left(s_{i,init}\right)}_{T}={\left({\Phi_{i}}_{T}\right)}^{†}r_{i},{\left(x_{i,init}\right)}_{T^{c}}=0$ 4: $r_{i,res}=r_{i}-\Phi_{i}s_{i,init}$ 5: $\beta_{i}^{0}=0$ 6: Res=ε+1 7: **while** $t<T_{\max}$ and Res>ε **do** 8:  $\hat{\beta_{i}^{t}}=\beta_{i}^{t}+\mu \left[\Phi_{i}^{H}\left(r_{i}-\Phi_{i}\beta_{i}^{t}\right)\right]$ 9:  $\lambda =|\hat{\beta_{i}^{t}}{|}_{K+1}/\mu$10:  $\beta_{i}^{t+1}=f_{\lambda \mu }(\beta_{i}^{t}+\mu \left[\Phi_{i}^{H}\left(r_{i}-\Phi s_{i,res}^{t})\right]\right)$11:  $Res=∥\beta_{i}^{t+1}-\beta_{i}^{t}{∥}_{2}$12:  t=t+113: **end while**
14: ${\hat{s}}_{i}=\beta_{i}^{t+1}+x_{i,\mathrm{init}}$15: $T^{\prime }=\mathrm{supp}\left({\hat{s}}_{i}\right)$16: $s_{i}={{\Phi_{i}}_{T^{\prime }}}^{†}y_{i}$17:
**end for**
18:
$s\left(x,y\right)=\max_{i}\left|s^{i}\left(x,y\right)\right|$
**Output:** s(x,y)


In WASAR imaging, it will cost huge amount of memory to store the measurement matrix. The azimuth-range decouple operators can be used to reduce the memory cost [5]. In this paper, we take BP based operators to substitute the measurement matrix and its conjugate transpose in real WASAR imaging. With the BP based operators, the memory cost can be reduced dramatically. If we reconstruct the measurement matrix, the memory cost is O(PQMN). With the BP based operators, the memory cost is O(MN). It means that, with the measurement matrix, the memory cost is reduced from $O(n^{2})$ to O(n).

BP mainly includes two operations: range Fourier transform and azimuth coherent addition. The imaging and raw data generation procedures are formulated as

(21) $$I\left\{\cdot \right\}≅R^{-1}\left\{H\left\{F^{-1}\left\{R\left\{\cdot \right\}\right\}\right\}\right\},$$

(22) $$G\left\{\cdot \right\}≅R\left\{F\left\{H^{-1}\left\{R^{-1}\left\{\cdot \right\}\right\}\right\}\right\},$$

where F and $F^{-1}$ are the the Fourier transform pairs, H is azimuth coherent addition operator and ${\left(H\right)}^{-1}$ is its inverse operation, R reshapes the vector into matrix and $R^{-1}$ reshapes the matrix into a vector.

## 4. Real Data Experiment

In this section, we will use two datasets to show the effectiveness of the proposed method.

### 4.1. Turntable Data

The turntable data collected by the Institute of Electronics, Chinese Academy of Sciences will be used to show the effectiveness of the proposed method. The real data of a metal tank model are measured in an anechoic chamber on a turntable, which is in uniform circular motion. The radar is a stepped frequency type and has a center frequency of 15 GHz and bandwidth 6 GHz. The turntable plane and its center are set as the imaging ground plane and the coordinate origin, respectively. The radius of equivalent circular passes is 8.54 m. The $360^{\circ }$ whole aperture is divided into 36 subapertures. The pixel size of the SAR image is 0.25 cm × 0.38 cm.

**Figure 2:**
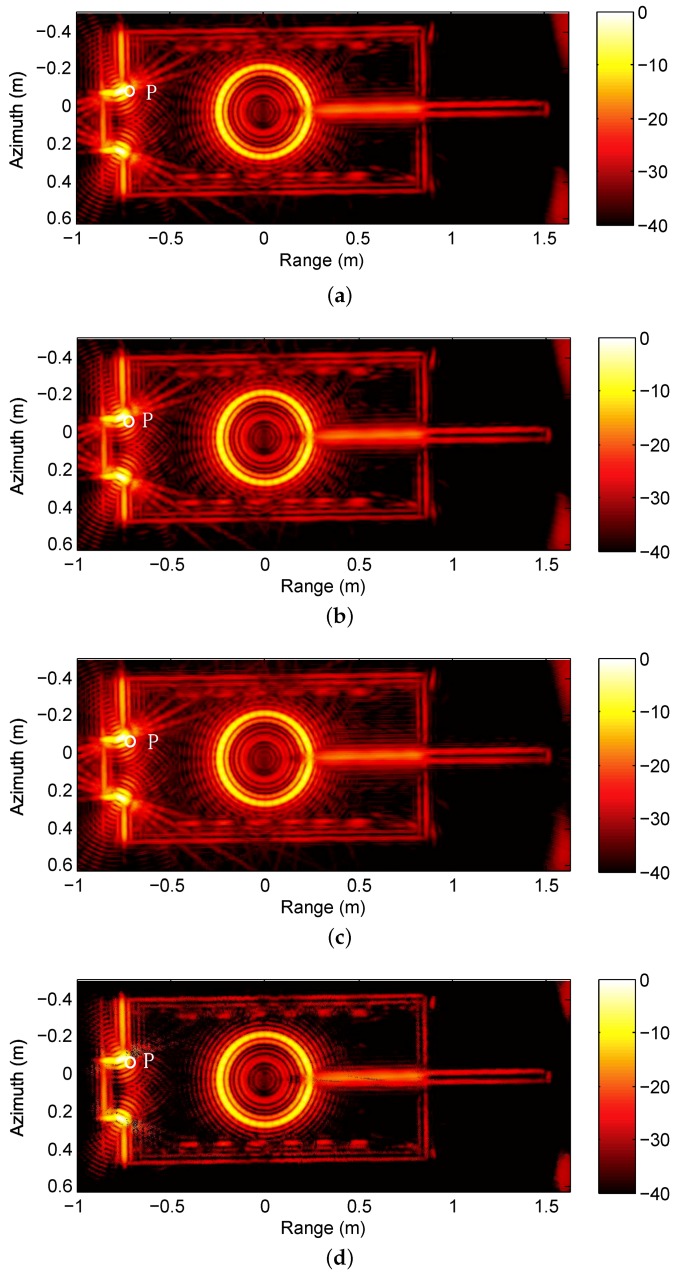
Results of the four methods. (**a**) GLRT result of BP; (**b**) GLRT result of CS; (**c**) GLRT result of debiased-CS; (**d**) GLRT result of LS-CS-Residual.

We reconstruct the subaperture images with BP, CS, debiased-CS and LS-CS-Residual. The results of the three methods are shown in Figure 2. Figure 2a is the result of BP, which is used as the referenced image. Compared with the result of the three method, the result of LS-CS-Residual remains less artifects as shown in the white circle.

**Figure 3:**
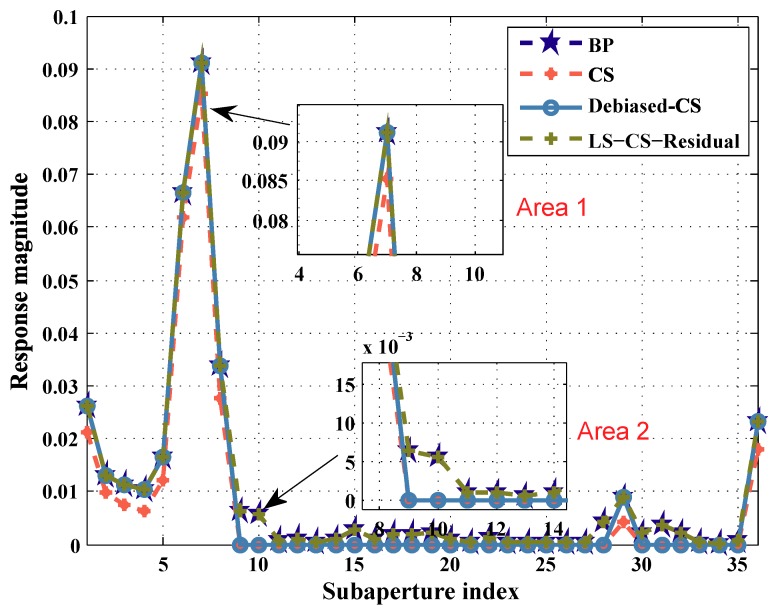
Reconstructed aspect dependent scattering of pixel P via the three methods.

To compare the performance of the three methods in the reconstruction of aspect dependent scattering, we select an aspect dependent scattering target P and plot its aspect dependent amplitude curve Figure 3. The result of BP is used as the reference. In Figure 3, we select Area 1 to show the performance of LS-CS-Residual to reduce the underestimation. Area 2 in Figure 3 is selected to show the performance of LS-CS-Residual to reduce the missed detections. As shown in Figure 3 Area 1, CS underestimates the amplitude of the target. The results of Debiased-CS and LS-CS-Residual highly overlap the result of BP. So Debiased-CS and LS-CS-Residual avoid the underestimation caused by CS. In Figure 3 Area 2, CS and debiased-CS fail in reconstructing the weak scattering. Since the support information of the other subapertures is adopted in LS-CS-Residual, the support of weak scattering target is preserved in the subapertures. So with the prior support information and the final debiasing step, LS-CS-Residual can reconstruct the aspect dependent scatterings of the targets more accurately than CS and debiased-CS.

The time taken by the three algorithms is given in the Table 1. Debiased-CS takes more time because of the debias step compared with CS. Compared with the former two algorithms, LS-CS-Residual takes similar amount time.

### 4.2. Gotcha Volumetric SAR DATA

Gotcha volumetric SAR dataset [22] is X-band circular SAR data that consists of CSAR phase history data collected at the X-band with a 640-MHz bandwidth. The spotlighted scene is a parking lot in an urban environment. The scene consists of numerous civilian vehicles and reflectors.

**Figure 4:**
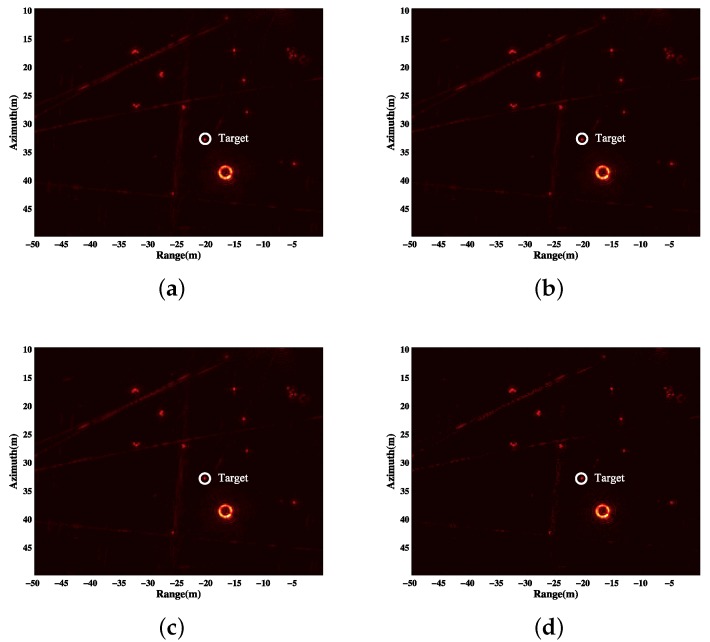
Results of the four methods. (**a**) GLRT result of BP; (**b**) GLRT result of CS; (**c**) GLRT result of debiased-CS; (**d**) GLRT result of LS-CS-Residual.

In this experiment, the HH polarization data are used. The whole aperture of $360^{\circ }$ is divided into 180 subapertures. Every subaperture is $4^{\circ }$. The apertures overlap every $2^{\circ }$. The pixel size is 0.2 m × 0.2 m. The area of reflectors is chosen. We reconstruct the scene with BP, CS, debiased-CS and LS-CS-Residual. The GLRT results of the four methods are shown in Figure 4.

**Figure 5:**
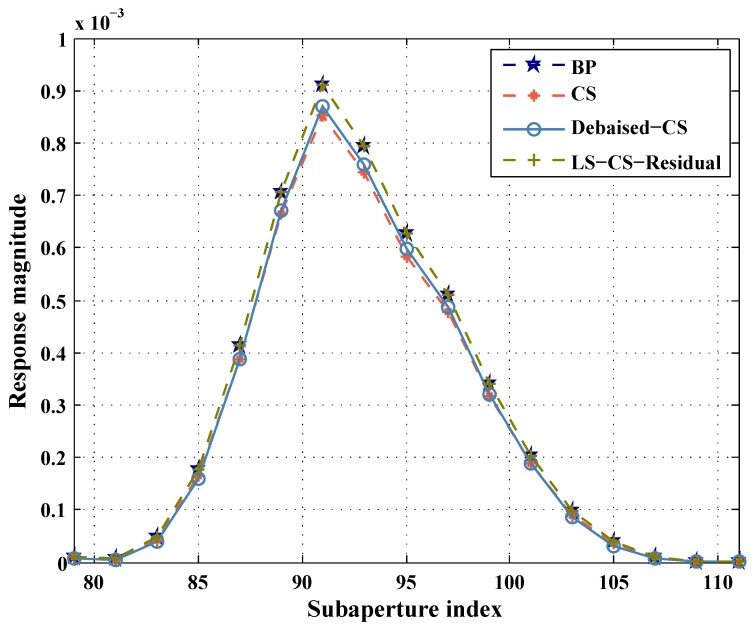
Reconstructed aspect dependent scattering of target via the three methods.

To evaluate the aspect dependent reconstruction performance of different methods, we select an aspect dependent scattering target and plot its reconstructed aspect dependent scattering. The selected target is a reflector that distributes across several pixels. We reconstruct the subaperture images with BP, CS, debiased-CS and LS-CS-Residual. To compare the aspect dependent scattering reconstruction performance of the three methods, we add the intensities of these pixels together and plot the results in Figure 5. Figure 5 is the main valid scattering area of the reflector. BP result is used as the reference. It is shown that the result of LS-CS-Residual is highly overlapped with the results of BP. The intensities of CS and debiased-CS are less than BP and LS-CS-Residual. The underestimation of debiased-CS is mainly caused by the missed detections. Since there are some missed detections in the result of debiased-CS, the intensities of the debiased-CS is less than BP. CS causes bias and missed detections because of the regularizer term. With the bias and missed detections, the peak of CS is lower than the other three methods.

The time taken by the three algorithms is given in Table 2. Table 2 shows that the three algorithms take similar amounts of time.

## 5. Conclusions

In this paper, an accurate WASAR imaging algorithm based on LS-CS-Residual is proposed. The traditional regularized subaperture imaging method based on $L_{1}$ regularization introduces the bias and missed detections which will cause inaccurate aspect dependent scattering estimates. To overcome this problem, LS-CS-Residual has been introduced into WASAR imaging. LS-CS-Residual mainly has three steps: initial LS estimate, CS on the residual and final LS estimate. The LS estimate step can be used to reduce the bias. The missed detections are reduced because the support information is adopted in the process of the LS-CS-Residual. The proposed method accommodates aspect dependent scattering better than CS and debiased-CS. The experiment results demonstrate its validity. 

## Figures and Tables

**Table 1:** Time taken (in minutes) by the three algorithms.

CS	Debiased-CS	LS-CS-Residual
22.31	23.43	20.08

**Table 2:** Time taken (in minutes) by the three algorithms.

CS	Debiased-CS	LS-CS-Residual
154.94	162.69	139.45

## Abbreviations

The following abbreviations are used in this manuscript:

SAR	Synthetic aperture radar
WASAR	Wide angle synthetic aperture radar
CSAR	Circular synthetic aperture radar
LS	Least squares
CS	Compressed sensing
LS-CS-Residual	Least squares on compressed sensing residual
BP	Backprojection
CS-Residual	CS on the residual
ISTA	Iterative shrinkage thresholding algorithm
GLRT	Generalized likelihood ratio test
